# Asthma Patients Benefit More Than Chronic Obstructive Pulmonary Disease Patients in the Coronavirus Disease 2019 Pandemic

**DOI:** 10.3389/fmed.2021.709006

**Published:** 2021-09-10

**Authors:** Ruoyan Xiong, Zhiqi Zhao, Huanhuan Lu, Yiming Ma, Huihui Zeng, Yan Chen

**Affiliations:** ^1^Department of Pulmonary and Critical Care Medicine, The Second Xiangya Hospital of Central South University, Changsha, China; ^2^Research Unit of Respiratory Diseases, Central South University, Changsha, China; ^3^Hunan Centre for Evidence-Based Medicine, Changsha, China

**Keywords:** COVID-19, pandemic, asthma, chronic obstructive pulmonary disease, management

## Abstract

**Background:** Coronavirus disease 2019 (COVID-19) has raised many questions about the role of underlying chronic diseases on disease outcomes. However, there is limited information about the effects of COVID-19 on chronic airway diseases. Therefore, we conducted the present study to investigate the impact of COVID-19 on patients with asthma or chronic obstructive pulmonary disease (COPD) and ascertain risk factors for acute exacerbations (AEs).

**Methods:** This single-center observational study was conducted at the Second Xiangya Hospital of Central South University, involving asthma or COPD patients who had been treated with inhaled combination corticosteroids (ICSs), such as budesonide, and one long-acting beta-2-agonist (LABA), such as formoterol, for at least a year before the COVID-19 pandemic. We conducted telephone interviews to collect demographic information and clinical data between January 1, 2019, and December 31, 2020, focusing on respiratory and systemic symptoms, as well as times of exacerbations. Data for asthma and COPD were then compared, and the risk factors for AEs were identified using logistic regression analysis.

**Results:** A total of 251 patients were enrolled, comprising 162 (64.5%) who had asthma and 89 who had COPD, with none having COPD/asthma overlap. Frequency of AEs among asthma patients was significantly lower in 2020 than in 2019 (0.82 ± 3.33 vs. 1.00 ± 3.16; *P* < 0.05). Moreover, these patients visited the clinic less (0.37 ± 0.93 vs. 0.49 ± 0.94; *P* < 0.05) and used emergency drugs less (0.01 ± 0.11 vs. 007 ± 0.38; *P* < 0.05) during the COVID-19 pandemic. In contrast, among COPD patients, there were no significant differences in AE frequency, clinic visits, or emergency drug use. Furthermore, asthma patients visited clinics less frequently during the pandemic than those with COPD. Logistic regression analysis also showed that a history of at least one AE within the last 12 months was associated with increased AE odds for both asthma and COPD during the COVID-19 pandemic (odds ratio: 13.73, 95% CI: 7.04–26.77; *P* < 0.01).

**Conclusion:** During the COVID-19 pandemic, patients with asthma showed better disease control than before, whereas patients with COPD may not have benefited from the pandemic. For both diseases, at least one AE within the previous 12 months was a risk factor for AEs during the pandemic. Particularly, among asthma patients, the risk factors for AE during the COVID-19 pandemic were urban environment, smoking, and lower asthma control test scores.

## Introduction

Coronavirus disease 2019 (COVID-19) is an acute respiratory infectious disease caused by severe acute respiratory syndrome coronavirus 2 (SARS-CoV-2). By March 30, 2021, the ongoing pandemic was reported to have a global morbidity of more than 126 million and a global mortality of over 2.7 million deaths (1). Given that COVID-19 mainly spreads via respiratory droplets during close contact (2) and SARS-CoV-2 can infect both the upper and lower airways (3), the roles of existing chronic airway diseases on COVID-19 outcomes have received much attention (4–7). However, limited information is available about the impact of the COVID-19 pandemic on chronic airway disease patients.

Of these chronic airway diseases, asthma and chronic obstructive pulmonary disease (COPD) are the most common worldwide, reporting a global morbidity of more than 330 million in asthma and 251 million in COPD (8, 9). Asthma is defined as a non-communicable disease characterized by airway inflammation, airway hyper-responsiveness, variable airflow limitation, and airway remodeling (10). On the other hand, chronic obstructive pulmonary disease (COPD), which is known as the third leading cause of death globally (11), is a common chronic airway disease characterized by constant respiratory symptoms and airflow limitation (12). Although epidemiological studies have shown no increased risk of COVID-19 morbidity or severity in patients with asthma (13), COPD was found to seemingly increase the risk of death in COVID-19 patients (14, 15). Moreover, a cohort study including 8.3 million (16) people reported that COPD was an independent risk factor for hospital admission.

With the spread of the COVID-19 pandemic, strict protective measures, including social distancing, mask wearing, and home quarantine, have been implemented to reduce SARS-CoV-2 transmission. As asthma and COPD exacerbations can both be triggered by respiratory infections and air pollution, among others (12, 17), COVID-19 and its resulting social isolation measures may have had effects on chronic airway disease patients in several ways. Since previous studies have indicated that patients with asthma or COPD experienced improved disease control during the pandemic due to behavioral changes (18–21), we hypothesized that the COVID-19 pandemic would confer an impact on the conditions of chronic respiratory diseases patients. Therefore, the present study aimed to compare the differences in clinical characteristics before and during the pandemic between asthma and COPD patients to analyze their risk factors for acute exacerbations (AEs) during the pandemic, and further to develop management strategies for chronic airway disease patients.

## Patients and Methods

### Study Design

**Figure 1:**
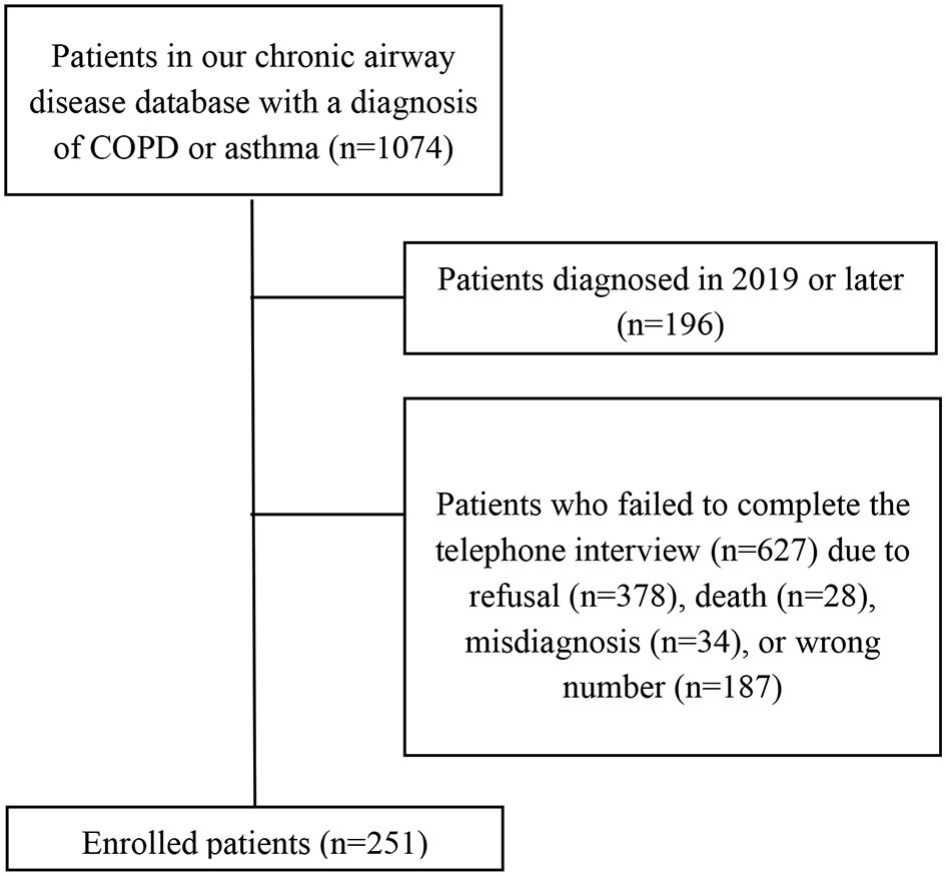
Flow chart of the follow-up process. Refusal refers to refusal to call or interview or no answer to call.

This study was a single-center observational survey performed at the Second Xiangya Hospital of Central South University and was approved by the Medical Research Ethics Committee of the Second Xiangya Hospital of Central South University. Patients were selected from the hospital records database, using the inclusion criteria as follows: (1) at least 2 years since COPD diagnosis according to the Global Initiative for Chronic Obstructive Lung Disease (GOLD) Report (22), or asthma diagnosis according to the Global Initiative for Asthma (GINA) Guidelines (10); and (2) history of using inhaled combination corticosteroids (ICSs), such as budesonide, and one long-acting beta-2-agonist (LABA), such as formoterol, for at least 1 year before January 1, 2020. Meanwhile, patients who refused to answer calls or co-operate with interviews were excluded from the study (Figure 1). All enrolled COPD patients were noted to have met the ICS/LABA indications, and informed written consent was signed by all participants. Afterwards, demographic and clinical data were collected from database records and routine follow-ups, and telephone interviews were conducted between January 5 and January 15, 2021 to collect clinical data between January 1, 2019, and December 31, 2020, focusing on respiratory and systemic symptoms, and times of exacerbations, among others. Collected data for asthma and COPD patients were then compared, and the risk factors for AEs were explored using logistic regression analysis.

### Data Collection

Data were collected in telephone interviews between January 5 and January 15, 2021. Interviewers participating in this study were required to have undergone centralized training. After obtaining approval from the participants, the interviewers asked them questions from a designed case report form.

### Demographic Characteristics and Clinical Data

Demographic characteristics included age, sex, occupation, residence, smoking history, and history of dust exposure. Clinical data from 2019, prior to the COVID-19 pandemic, comprised history of COPD or asthma, number of AEs, emergency drug use due to AEs, and the number of online consultations or clinic visits. In addition, the following clinical data were collected in 2020, during the COVID-19 pandemic, including number of AEs, emergency drug use due to COPD or asthma AEs, and the number of online consultations and clinic visits. Specifically, the modified Medical Research Council (mMRC) dyspnea scale (23) and the asthma control test (ACT) (24) were used to assess the symptoms of COPD and asthma, respectively. In these cases, AEs were referred to as AECOPDs in COPD patients, according to the GOLD Report, (22) or AEs of asthma, according to the GINA Guidelines (10).

### Statistical Analysis

Continuous variables were expressed as means ± standard deviation or as medians (interquartile range [IQR]), whereas categorical variables were expressed as numbers (lrb%). The significance of differences were assessed by paired-sample or independent-sample *t* test for continuous variables and by the χ^2^ test for categorical variables. Association between risk factors and asthma/COPD AEs was examined by multivariable logistic regression analyses. The SPSS software (version 23.0) was used for all analyses, and results were considered statistically significant at P < 0.05.

## Results

Patients from our database who met the inclusion criteria were contacted by telephone. Of the 878 contacted patients, 627 were excluded due to refusal to call or interview, death, misdiagnosis, or wrong numbers, ultimately enrolling 251 patients. Of these, 162 (64.5%) had asthma, 89 had COPD, and none had a COPD and asthma overlap. No severe infections or COVID-19 cases were also observed among the included patients. Median age was noted to be 56 years (IQR: 47–63 years), and 55.0% of all patients were male, comprising 74 of the 162 asthma patients (45.7%) and 64 of the 89 COPD patients (71.9%). Regarding risk factors for AEs, ~10% of all patients had a history of dust exposure, 33.5% (84) were former or current smokers, and ~60% of them lived in urban areas. Demographic characteristics are further detailed in Tables 1, 2.

**Table 1:** Demographic Characteristics of the included patients (*N* = 251).

**Characteristic**	**Category**	**Median or N (%)**
Total		251
Age (years, 18–81years)		56 (47, 63)
Sex	Male	138 (55.0)
Residence	Urban	154 (61.4)
Dust exposure	Former or current^#^	25 (10.0)
Smoking	Former or current	84 (33.5)
Diagnosis	Asthma	162 (64.5)

*Data are expressed as medians (interquartile range) or as N (%)*.

# *, Patients who were firework factory workers, coal mine workers, welders, or builders*.

*COPD, chronic obstructive pulmonary disease*.

**Table 2:** Comparisons of demographic characteristics between asthma and COPD patients.

**Variables**	**Asthma (*n* = 162)**	**COPD (*n* = 89)**	***P***
Age (years)	51.60 ± 13.18	59.96 ± 9.78	<0.01
Sex (male)	74 (45.7%)	64 (71.9%)	<0.01
Residence (rural)	57 (35.2%)	40 (44.9%)	0.13
Dust exposure (former or current)	13 (8.0%)	12 (13.5%)	0.17
Smoker (former or current)	14 (8.6%)	11 (12.4%)	0.35

*Data expressed as n (%) or as means ± standard deviation*.

*COPD, chronic obstructive pulmonary disease*.

The number of AEs was reported to be lower in 2020 than in 2019 (0.86 ± 2.97 vs. 1.00 ± 2.85; *P* < 0.05). Similarly, the number of clinic visits and emergency drug use also decreased, which persisted even when asthma patients were analyzed separately. Specifically, patients with asthma suffered fewer AEs (0.84 ± 3.34 vs. 1.00 ± 3.16; *P* < 0.01), visited the hospital less frequently (0.37 ± 0.93 vs. 0.49 ± 0.94; *P* < 0.05), and had a decreased frequency of emergency drug use in 2020 as compared to that of 2019 (0.30 ± 1.42 vs. 2.05 ± 6.36; P < 0.05). Additionally, asthma patients scored higher on the ACT in 2020 than that in 2019 (22.18 ± 3.13 vs. 21.20 ± 3.60; P < 0.01); however, no demographic differences in the clinical information of COPD patients were observed between 2020 and 2019 (Table 3). Given these findings, it seemed that the COVID-19 pandemic had a greater impact on patients with asthma than on those with COPD.

**Table 3:** Comparisons of patients' conditions between asthma and COPD.

**Variables**	**Total**			**Asthma**			**COPD**			***P* ^#^**
	**2019**	**2020**	***P***	**2019**	**2020**	***P***	**2019**	**2020**	***P***	
Frequency of AEs (/year)	1.00 ± 2.85	0.86 ± 2.97	0.01	1.00 ± 3.16	0.84 ± 3.34	<0.01	1.01 ± 2.19	0.93 ± 2.16	0.47	0.20
Frequency of clinic visits (/year)	0.61 ± 1.17	0.46 ± 0.95	<0.01	0.49 ± 0.94	0.37 ± 0.93	<0.01	0.16 ± 0.51	0.62 ± 0.96	0.31	0.03
Frequency of emergency drug use (/year)	1.36 ± 5.15	0.23 ± 1.16	<0.01	2.05 ± 6.36	0.30 ± 1.42	<0.01	0.16 ± 0.51	0.12 ± 0.50	0.16	0.78
ACT score				21.20 ± 3.60	22.18 ± 3.13	<0.01				
mMRC							1.44 ± 1.24	1.41 ± 1.22	0.44	

*Data are expressed as means ± standard deviation*.

*P^#^, Comparisons of patients' conditions in 2020 between asthma and COPD*.

*ACT, asthma control test; AE, acute exacerbation; COPD, chronic obstructive pulmonary disease*.

Differences in conditions between COPD and asthma patients were also studied. Regarding clinic visits, patients with asthma visited the clinic less frequently than those with COPD in 2020 (0.37 ± 0.93 vs. 0.62 ± 0.96; *P* < 0.05). Conversely, frequencies of AEs and emergency drug use were not significantly different in between COPD and asthma patients (Table 3). Furthermore, there were no significant differences between the patients living in urban areas and those living in rural areas, with regards to the frequency of AEs, clinic visits, and emergency drug use (Supplementary Table).

To determine the potential risk factors for AEs during the pandemic, the patients were divided into two groups: (1) an AE group, comprising patients who had at least one AE in 2020, and (2) a non-AE group, comprising patients who had no AEs in 2020. Comparisons of the proportions and correlation analyses were performed using the chi-squared test between these two groups. In the AE and non-AE groups, 65.7% and 50.8% of the patients were male, respectively (*P* < 0.05). Moreover, the AE group had a greater proportion of patients with a history of dust exposure (18.6 vs. 6.6%; *P* < 0.01), and 51.4% of the AE group patients were former or current smokers, which was markedly higher than in the non-AE group (26.5%). Additionally, the proportion of patients who had an AE in 2019 was higher in the AE group (72.9 vs. 16.0%; *P* < 0.01), which persisted even when we analyzed asthma patients separately from those with COPD (Table 4). In addition to sex, dust exposure, smoking history, and AE in 2019, the residence distribution was also different between the two groups, wherein more rural residents in the AE group were noted as compared to the non-AE group. Furthermore, the AE group had lower ACT scores than the non-AE group, and among the COPD patients, the AE group had higher mMRC scores than the non-AE group. These details are further listed in Table 4.

**Table 4:** Comparisons between AE and non-AE patients during the COVID-19 pandemic (2020).

**Variable**	**Total**			**Asthma**			**COPD**		
	**AE**	**Non-AE**	***P*-Value**	**AE**	**Non-AE**	***P*-Value**	**AE**	**Non-AE**	***P*-Value**
*N*	70	181		42	120		28	61	—
Sex (male)	46 (65.7%)	92 (50.8%)	0.03	26 (61.9%)	48 (40%)	0.01	20 (71.4%)	44 (72.1%)	0.95
Residence (rural)	33 (47.1%)	64 (35.4%)	0.09	21 (50.0%)	36 (30.0%)	0.02	12 (42.9%)	28 (45.9%)	0.79
Age (>60)	40 (57.1%)	120 (66.3%)	0.18	27 (64.3%)	91 (75.8%)	0.15	13 (46.4%)	29 (47.5%)	0.92
Dust exposure (former or current)	13 (18.6%)	12 (6.6%)	<0.01	14 (33.3%)	12 (10.0%)	<0.01	6 (21.4%)	6 (9.8%)	0.14
Smoker (former or current)	36 (51.4%)	48 (26.5%)	<0.01	20 (47.6%)	9 (7.5%)	<0.01	12 (42.9%)	26 (42.6%)	0.98
Diagnosis (asthma)	42 (60.0%)	120 (65.7%)	0.40						
AE in last 12 months	51 (72.9%)	29 (16.0%)	<0.01	29 (69.0%)	20 (16.7%)	<0.01	22 (78.6%)	9 (14.8%)	<0.01
ACT				20.15 ± 3.20	22.88 ± 2.75	<0.01			
mMRC							2.18 ± 1.19	1.05 ± 1.06	<0.01

*Data were expressed as n (%) or means ± standard deviation*.

*ACT, asthma control test; AE, acute exacerbation; COPD, chronic obstructive pulmonary disease; mMRC, modified Medical Research Council*.

Binary logistic regression was used to assess possible risk factors, showing that the proportion or mean was significantly different between the AE and non-AE groups. Among all chronic airway disease patients, the final logistic model revealed two risk factors: dust exposure and AE in 2019. Specifically, patients with a history of dust exposure had a 2.91-fold higher risk of AE than those without, and a history of at least one AE in 2019 was also associated with increased odds of AE in this year (odds ratio [OR]: 13.73; 95% confidence interval [CI]: 7.04–26.77; *P* < 0.01). Among patients with asthma, rural patients had a lower risk of AE than those living in urban areas (OR: 0.36, 95% CI: 0.14–0.94; *P* < 0.05). In addition, smoking (OR: 3.34, 95% CI: 1.27–8.74; *P* < 0.05), history of AE in 2019 (OR: 8.93, 95% CI: 3.56–22.39; *P* < 0.001), and lower ACT scores (OR: 4.32, 95% CI: 1.56–12.01; *P* < 0.01) were associated with increased odds of AE. On the other hand, in patients with COPD, only a history of AE in 2019 (OR: 18.22, 95% CI: 5.59–59.32; *P* < 0.01) was associated with increased odds of AE. More information on the logistic regression findings is provided in Table 5.

**Table 5:** Logistic regression analysis of risk factors related to AE.

**Variable**	**Total**		**Asthma**		**COPD**	
	**OR (95% CI)**	***P*-Value**	**OR (95% CI)**	***P*-Value**	**OR (95% CI)**	***P*-Value**
Residence (rural)			0.36 (0.14–0.94)	0.04		
Dust exposure (former or current)	2.91 (1.05–8.09)	0.04				
Smoker (former or current)			3.34 (1.27–8.74)	0.01		
AE in last 12 months	13.73 (7.04–26.77)	<0.01	8.93 (3.56–22.39)	<0.01	18.22 (5.59–59.32)	<0.01
ACT (<23)			4.32 (1.56–12.01)	<0.01		
mMRC (>1)					3.21 (0.99–10.43)	0.05

*Variables that significantly differed in the univariate analysis (Table 4) were included in the logistic regression analysis*.

*ACT, asthma control test; AE, acute exacerbation; COPD, chronic obstructive pulmonary disease; mMRC, modified Medical Research Council; OR, odds ratio*.

## Discussion

To date and to the best of our knowledge, this was the first study to analyze the differences between asthma and COPD patients during the COVID-19 pandemic. It was found that there was a decrease in the frequency of AEs, clinic visits, and emergency drug use in 2020 among asthma patients; however, this was not observed in COPD patients. Moreover, patients with asthma went to the clinic less frequently than those with COPD in 2020. The population in the present study had used combined budesonide and formoterol since 2018, which were maintained before and during the pandemic, indicating that drug bias was likely low. Furthermore, no severe infections or COVID-19 cases were found, also indicating that ICS-induced infection incidence was low. Given that previous studies (25–27) have found variable AE frequencies of asthma and COPD in different seasons, the present study collected data from January 1, 2019, to December 31, 2020, avoiding season bias in our comprehensive observation and analysis of data across two entire years.

In the present survey, the patients' conditions before and during the COVID-19 pandemic were evaluated. During the pandemic, asthma patients showed better disease control, with fewer AEs, fewer medical visits, lower emergency drug use frequencies, and higher ACT scores in 2020 than in 2019. This finding may be attributed to their less exposure to outdoor air pollutants (28, 29), allergens (30), and respiratory viruses (31, 32) as they practiced social isolation measures, including home quarantine, social distancing, and public health initiatives (33). Moreover, one study reported a significant decline in the concentration of air pollutants caused by control measures to mitigate the spread of COVID-19 during the pandemic (34). As such, the improved air quality may have helped stabilize the condition of asthma patients and reduce the frequency of AE. This is also affirmed by one multinational cohort study involving 1,054 children with asthma, showing that the outcomes of childhood asthma improved during the pandemic (18). Furthermore, research in the US and UK has shown a marked reduction in emergency visits by asthma patients during the pandemic (19, 35). However, it should be noted that past research has mainly focused on childhood asthma, whereas adults were mainly involved in the present study. Nevertheless, the aforementioned findings demonstrated that patients with asthma experienced improved conditions during the COVID-19 pandemic.

In patients with asthma, a history of at least one AE in the last 12 months was determined as a risk factor for AE during the COVID-19 pandemic, which is consistent with previous findings (36). Logistic regression analysis revealed that rural environments could be a protective factor in asthma patients, perhaps because there are lower concentrations of outdoor air pollutants in rural areas than in urban areas in China (37), given that outdoor air pollution, including particulate matter and gaseous pollutants, has already been associated with asthma prevalence and AEs (38, 39). The present results also supported the prior finding (40) that cigarette smoking and lower ACT scores were risk factors for AE.

Meanwhile, in patients with COPD, the frequency of AEs, medical visits, and emergency drug use before the pandemic did not differ significantly from those during. In contrast with our findings, Faria et al. (20) reported a 73.4% decrease in severe AECOPD events in the first half of 2020 as compared to the same timeline of previous years, whereas Tan et al. (21) found that AECOPD admissions decreased by more than 50% over a sustained 6-month period in 2020 as compared to previous years. These discrepancies may have arisen due to the different drugs usedm or these previous studies may have shown season bias due to insufficient observation. In the COPD patients from our study, only two variables were included in the final logistic model. Additionally, even though mMRC was included, no significant differences were found between the two groups (*P* = 0.05). Although previous studies have shown that smoking was one of the most common reasons for COPD prevalence and exacerbations (12), it was not included in the final logistic model. However, it should also be noted that only 89 COPD patients were included in the present study, so the sample size seems limited for logistic regression analysis.

In the present study, COPD patients were found to be older than those with asthma, possibly due to the difference in disease onset (12, 17). In addition, the proportion of males was higher among COPD patients, which was likely because the smoking rate was lower among females (41). There were also significant differences in the clinical data between asthma and COPD patients. Specifically, the frequencies of AE, clinic visits, and emergency drug use decreased in 2020 among patients with asthma, whereas no significant differences were observed in patients with COPD. These findings seemed to show that the outcomes in asthma patients improved during the COVID-19 pandemic, while those in COPD patients did not, which was perhaps due to the differences in the risk factors for the progression and exacerbation of the two diseases. Although social isolation measures were found to reduce exposure to respiratory viruses, which play an important role in both asthma and COPD AEs (31, 32), prolonged exposure to indoor air pollution during home quarantine may increase the risk of AE among COPD patients. In fact, studies have shown that excessive passive smoking and harmful fumes from kitchens during home quarantine are closely related to COPD prevalence and exacerbations (42–45). As nearly half of the COPD patients in the present study were rural residents, fuels may have adverse effects on disease control during the pandemic, given that coal and wood are still widely used for cooking in Chinese rural areas (46). The China Pulmonary Health [CPH] study reported an overall COPD prevalence of 8.6% in Chinese population and higher prevalence in rural residents than in urban residents (9.6 vs 7.4%; *p* = 0.047) (47), but no significant difference in asthma prevalence was observed between rural and urban areas (4.9 vs 3.6%, *p* = 0.27) (38). Furthermore, recent studies (39) and the logistic regression of the present study indicated that outdoor air pollution played a significant role in asthma exacerbations and that outdoor air pollution played a smaller role than smoking in COPD prevalence and exacerbations (48). Additionally, since the rate of rural residents was higher among asthma patients in this study, poorer access to health care facilities in rural areas might also explain the fewer frequency of clinical visits in 2020 in asthma patients than that in COPD patients. Nonetheless, asthma patients still showed significant benefits with fewer AEs and higher ACT scores. This may demonstrate that personal protection and social distance ensured a better disease control for asthma patients during the pandemic.

Taken together, patients with asthma may be more sensitive to outdoor air pollution and allergens than those with COPD, indicating that home quarantine and mask-wearing were sufficient for exacerbation prevention and control in these patients during the COVID-19 pandemic. Although there is a need to conduct multi-centers and larger-sample study, we speculate that there are different effects of indoor or outdoor air pollutions and various personal protections on asthma and COPD patients in future potential pandemics. On the other hand, the result of our study supported personal protection and social distance during pandemic not only for communicable disease, but also for the management of chronic airway diseases. Additionally, due to a worse control of COPD patients in our study, we suggest that physicians should pay more attention to COPD patients in future potential pandemics.

Despite these findings, we admit that there were certain limitations in our study. Firstly, the single and limited data source made the research less credible and reproducible as compared to a multi-center research study. However, with regard to telephone interviews, a single-center research will better avoid bias caused by the unevenness of the interviewer's level between different centers. Secondly, the patients' self-reported data may have affected the accuracy of the follow-up results due to subjective factors or memory bias. To mitigate this, we chose two internationally recognized symptom assessment scales, the modified Medical Research Council (mMRC) dyspnea scale and the asthma control test (ACT), to help us understand the symptoms and conditions of our patients. Interviewers participating in the study were also required to undergo centralized training. Thirdly, 627 patients were excluded due to refusal to call or interview, death, misdiagnosis, or wrong numbers, which may have led to follow-up bias to our results. This relatively high rate of loss to follow-up may have been caused by the people's increasing vigilance against phone scams and changing mobile phone numbers. Furthermore, since people who died before we conducted telephone interviews were excluded, we may have lost data from those with severe symptoms, which would have affected the representativeness and reliability of our research. Fourthly, only 89 COPD patients were enrolled in the present study, and this small sample size may have reduced the reliability of the data. Fifthly, since most of the patients were adults, our study lacked data on children and teenagers. Lastly, population in this study used only budesonide/formoterol, and thus outcomes may differ in patients who use other ICS/LABA regimens.

## Conclusion

During the COVID-19 pandemic, patients with asthma showed better disease control than before, whereas those with COPD did not. Among the population that used ICS/LABA, at least one AE within the previous 12 months was a risk factor for AE during the pandemic in both asthma and COPD patients. For patients with asthma, urban environment, smoking, and lower ACT scores were also risk factors for AE during the COVID-19 pandemic.

## Data Availability Statement

The original contributions presented in the study are included in the article/Supplementary Material, further inquiries can be directed to the corresponding author.

## Ethics Statement

The studies involving human participants were reviewed and approved by Medical Research Ethics Committee of the Second Xiangya Hospital of Central South University. Written informed consent for participation was not required for this study in accordance with the national legislation and the institutional requirements. Written informed consent was obtained from the minor(s)' legal guardian/next of kin for the publication of any potentially identifiable images or data included in this article.

## Author Contributions

All authors contributed to data analysis, drafting and revising the article, gave final approval of the version to be published, and agree to be accountable for all aspects of the work.

## Funding

This work was supported by National Natural Science Foundation of China (No.81873410, No.82070049, and No.81400032), National Key R&D Program of China (2016YFC1304700).

## Conflict of Interest

The authors declare that the research was conducted in the absence of any commercial or financial relationships that could be construed as a potential conflict of interest.

## Publisher's Note

All claims expressed in this article are solely those of the authors and do not necessarily represent those of their affiliated organizations, or those of the publisher, the editors and the reviewers. Any product that may be evaluated in this article, or claim that may be made by its manufacturer, is not guaranteed or endorsed by the publisher.
