# Supplementary material for: Asthma Patients Benefit More Than Chronic Obstructive Pulmonary Disease Patients in the Coronavirus Disease 2019 Pandemic
Source: Front Med (Lausanne). 2021 Sep 10;8:709006. doi: 10.3389/fmed.2021.709006 (PMC8460914; doi:10.3389/fmed.2021.709006)
Supplement: Supplementary file 1 [file Table_1.docx]

**Supplementary Table.** Comparison of conditions between urban and rural patients during pandemic (2020)

| **Variables** |  | **Mean** | | ***P*-Value** |
| --- | --- | --- | --- | --- |
|  |  | **Urban** | **Rural** |  |
| **Frequency of AE (/year)** | Total | 0.95±3.58 | 0.75±1.58 | 0.61 |
|  | Asthma | 0.88±3.95 | 0.82±1.81 | 0.97 |
|  | COPD | 1.16±2.70 | 0.65±1.19 | 0.27 |
| **Frequency of clinic visits (/year)** | Total | 0.46±1.03 | 0.46±0.81 | 1.00 |
|  | Asthma | 0.39±1.07 | 0.32±0.62 | 0.67 |
|  | COPD | 0.60±0.94 | 0.64±0.989 | 0.85 |
| **Frequency of Emergency drug use (/year)** | Total | 0.01±0.08 | 0.01±0.10 | 0.76 |
|  | Asthma | 0.30±1.42 | 0.14±0.61 | 0.70 |
|  | COPD | 0.16±0.62 | 0.07±0.27 | 0.87 |
| **ACT score** | Asthma | 22.11±3.20 | 22.30±2.94 | 0.70 |
| **mMRC** | COPD | 1.35±1.11 | 1.49±1.35 | 0.60 |

**Notes:** Data expressed as mean ± standard deviation.

**Abbreviations:** ACT, asthma control test; AE, acute exacerbation; COPD, chronic obstructive pulmonary disease; mMRC, modified Medical Research Council
